# Nanotechnology and natural killer cell immunotherapy: synergistic approaches for precise immune system adjustment and targeted cancer treatment in gastrointestinal tumors

**DOI:** 10.3389/fmed.2025.1647737

**Published:** 2025-08-21

**Authors:** Xiwen Kang, Danyang Li, Rui Sun

**Affiliations:** ^1^Department of Breast Surgery, The First Hospital of China Medical University, Shenyang, China; ^2^Department of Pharmacology, Harbin Medical University, Harbin, China; ^3^Department of Orthopedics, The First Hospital of China Medical University, Shenyang, China

**Keywords:** gastrointestinal cancers, NK cells, immunotherapy, gastrointestinal tumors, biomimetic nanocarriers, tumor targeting, stimuli-responsive systems, clinical translation

## Abstract

Gastrointestina (GI) tumors are a major contributor to global cancer-related illness and death, marked by their rapid growth, late detection, and resistance to standard treatments. NK cells, key cytotoxic components of the innate immune system, show promise in immunotherapy due to their ability to target tumor cells without requiring antigen presentation. Nonetheless, their effectiveness against gastrointestinal tumors is constrained by issues such as insufficient tumor penetration, *brief* survival in the body, and suppression by the immunosuppressive tumor microenvironment (TME). Meanwhile, nanotechnology has transformed cancer treatment by offering methods for precise drug delivery, immune system modulation, and improved bioavailability. Combining NK cells with specially designed nanoparticles (NPs) has created a powerful system with amplified cooperative anti-tumor effects, improving the targeting of tumors, activation of NK cells, and their endurance, while also allowing for control over the tumor immune microenvironment in both space and time. This thorough review investigates the complex interaction between the immunobiology of NK cells and the design of nanomaterials, specifically in the context of gastrointestinal tumors. Key areas of focus include nanoparticle-assisted engineering of NK cells, cytokine delivery, biomimetic disguise, reprogramming of the TME, and targeting of tumors specifically. We critically assess preclinical and emerging clinical evidence that supports the effectiveness of these combined therapies, analyze related safety and translational challenges, and suggest forward-looking approaches involving gene editing, innovative stimulus-responsive systems, and AI-assisted therapeutic personalization. In conclusion, this review presents the combined NK-NP approach as a promising new frontier in the advancement of personalized, immune-guided treatments for gastrointestinal cancers.

## 1 Introduction

Gastrointestinal (GI) malignancies, including colorectal, gastric, pancreatic, esophageal, and hepatocellular carcinomas, constitute a significant share of the global cancer burden. According to GLOBOCAN 2020 estimates, these cancers collectively account for more than 4.5 million new cases and approximately 3.3 million deaths annually, representing nearly 26% of cancer-related morbidity and mortality worldwide (1). Despite advances in diagnostics, surgical interventions, and chemotherapeutic regimens, GI tumors, especially in their metastatic forms, exhibit poor prognosis and low survival rates (2, 3). Main causes being high genetic heterogeneity, late-stage diagnosis, multidrug resistance, and a strong immunosuppressive tumor microenvironment (TME) that reduces the efficacy of both conventional and immune-based interventions (4).

**FIGURE 1:**
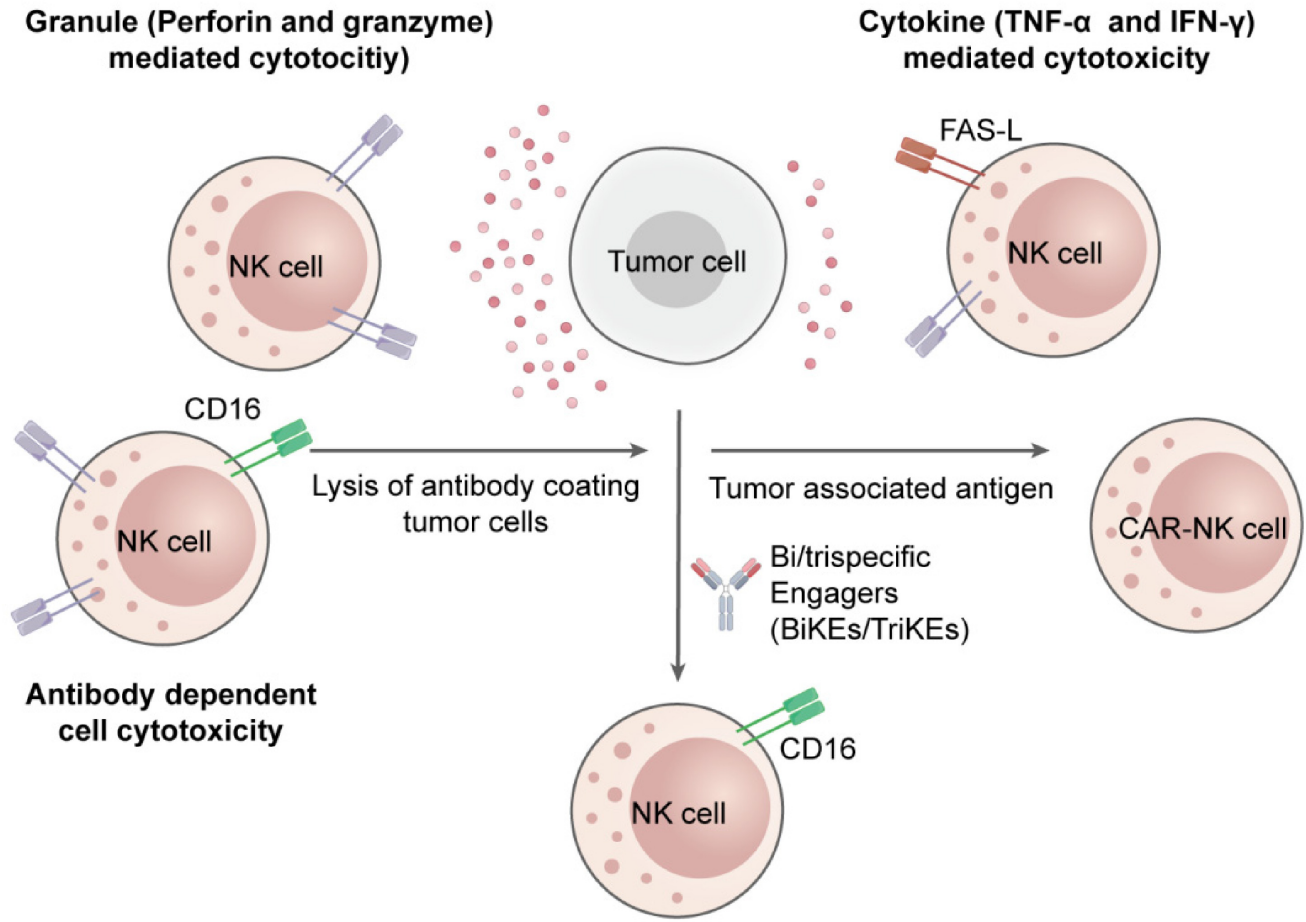
Mechanisms of NK cell cytotoxicity in suppressing tumor growth. NK cells induce tumor cell death through direct cytotoxic granule release, death receptor-mediated apoptosis, antibody-dependent cellular cytotoxicity, and secretion of cytokines to program the immune response and induce tumor regression.

Among inherent immune cells, NK cells have appeared as dominant effectors, which can target tumor cells independently of Major Histocompatibility Complex (MHC) restriction or antigen presentation (5). A fine-tuned balance between activating and inhibitory signals transmitted via germline-encoded surface receptors regulates their cytotoxic function. Once activated, NK cells exert tumor-suppressive effects through perforin-granzyme-mediated apoptosis, death receptor pathways, and secretion of pro-inflammatory cytokines such as interferon-gamma (IFN-γ) and tumor necrosis factor-alpha (TNF-α) (6, 7). Figure 1 displays these processes, which include NK immunological synapse formation, cytokine release pathways, and cytolytic granule polarization (8).

In spite of their natural cytotoxic properties, natural killer cells suffer significant challenges within the TME of gastrointestinal cancers. These challenges include the overexpression of checkpoint inhibitors PD-L1 and HLA-E. In addition, they face downregulation of activating ligands on cancerous tissue, and the release of suppressive cytokines such as transforming growth factor-beta (TGF-β) (9). Moreover, immunosuppressive cell populations like regulatory T cells (Tregs), myeloid-derived suppressor cells (MDSCs), and tumor-associated macrophages (TAMs) further compromise NK cell function. Structural features of the TME also limit viability and immune cell infiltration (10). At the same time, nanomedicine has resulted in the development of highly adaptable platforms that caused to increase therapeutic accuracy, improve targeted drug delivery, and boost the immune systems. Target oriented tailored (engineered) nanoparticles, including polymeric particles, liposomes, nanospheres, and inorganic systems, can encapsulate a wide-range of therapeutic agents (11). The surface of these nanoparticles can be functionalized with ligands to recognize tumor-specific markers and enhance selective accumulation in tumors. Besides, inactive accumulation has been used in various nanoparticle modulation for gastrointestinal tumor models (12, 13).

Integrating nanotechnology with NK cell-based immunotherapy offers a synergistic paradigm to address the limitations inherent to each modality when applied individually. Engineered nanoparticles can enhance NK cell efficacy by co-delivering immune checkpoint inhibitors (14). Additionally, nanoparticles cloaked with NK cell membranes exhibit tumor-specific targeting potentials while minimizing immune clearance. These multifunctional strategies can effectively remodel the tumor immune microenvironment (TIME), converting it into a more immune-stimulatory state that enables tumor eradication and activation (15).

Emerging technologies, including AI, stimuli-responsive nanocarriers, CRISPR-Cas9-mediated gene editing, and rational nanoparticle design, enable extraordinary accuracy in controlling therapeutic delivery (16, 17). These novelties help the synchronized activation of immune responses, real-time monitoring of NK cell dynamics, and spatially controlled release of therapeutic payloads. Advanced nanocarriers, responsive to tumor-specific environmental cues such as oxidative stress, pH shifts, and enzymatic activity, confirm targeted and efficient drug release at the tumor site (18).

This review presents a detailed analysis of NK cell cytotoxicity, regulating TIME, nanoparticle-mediated design strategies, and evolving clinical applications. Each section is validated with recent preclinical and clinical data and is accompanied by summaries, including the most relevant figures and tables. A forward-looking roadmap for future development, standardization, and personalization of NK-NP strategies is also proposed, based on integrating Good Manufacturing Practice (GMP), multi-omics profiling, AI-based optimization, and regulatory frameworks (19). This review’s scope is both foundational and futuristic. It outlines the immunobiological underpinnings of NK cells, the versatility of nanoparticle-based delivery systems, and the synergistic potential of their integration (20). Table 1 summarizes the significant challenges of NK cell therapies in GI tumors and the corresponding nanotechnology-based solutions, which will be elaborated in the subsequent sections.

**TABLE 1:** Challenges in NK cell therapy for GI tumors and their nanotechnology-based solutions.

NK cell therapy challenges	Nanotechnology-enabled strategies
**Limited tumor infiltration**	Chemokine-functionalized NPs, magnetic guidance, and membrane targeting
**Functional exhaustion in TME**	Co-delivery of checkpoint inhibitors (anti-NKG2A, anti-TIGIT) via NPs
**Short *in vivo* persistence**	Cytokine-loaded biodegradable NPs (e.g., IL-15, IL-21)
**Tumor heterogeneity and antigen loss**	Dual-acting NPs (cytokine-chimeric antigen receptor (CAR) ligand delivery, gene-editing payloads)
**Off-target immune activation**	Biomimetic NPs (e.g., NK membrane-cloaked silica or lipid-based platforms)

The following section discusses the mechanistic foundations of NK cell cytotoxicity, receptor regulation, and tumor microenvironment interactions. It lays the groundwork for understanding their therapeutic enhancement by integrating with recent nanotechnology applications.

## 2 Natural killer cells in tumor immunity

Natural killer cells are specialized lymphocytes of the innate immune system with the intrinsic capacity to recognize and eliminate transformed or virus-infected cells without prior sensitization. Unlike T cells, NK cells operate independently of classical antigen presentation, enabling them to mount rapid responses against malignancies (21). NK cells have the potency to work through both innate and adaptive immune interactions makes them interesting for tumor immunotherapy, including its effectiveness for GI cancer as well (22).

Despite robust effector functions, NK cells encounter significant suppression within the TME that limits their therapeutic capabilities. Therefore, before their utalization, a comprehensive understanding of their cytotoxicity mechanisms, receptor-mediated regulations, infiltration dynamics, and plans for functional enhancement is essential for their application in GI cancer immunotherapy (23).

### 2.1 Mechanisms of NK cell cytotoxicity

NK cell cytotoxicity is mediated through two well-established pathways: death receptor-induced apoptosis and granule exocytosis. After specification of target tumor cells, an immunological synapse facilitated by integrins (e.g., LFA-1) is formed by the NK cells. Cytotoxic granules containing granzymes and perforin are secreted within this synapse. Perforin forms pores in the target cell membrane, allowing granzymes to enter and activate caspase cascades. This results in mitochondrial damage and apoptosis (24). At the same time as, NK cells express death ligands, which bind to their relevant receptors on cancerous cells, persuading extrinsic apoptosis (7).

**FIGURE 2:**
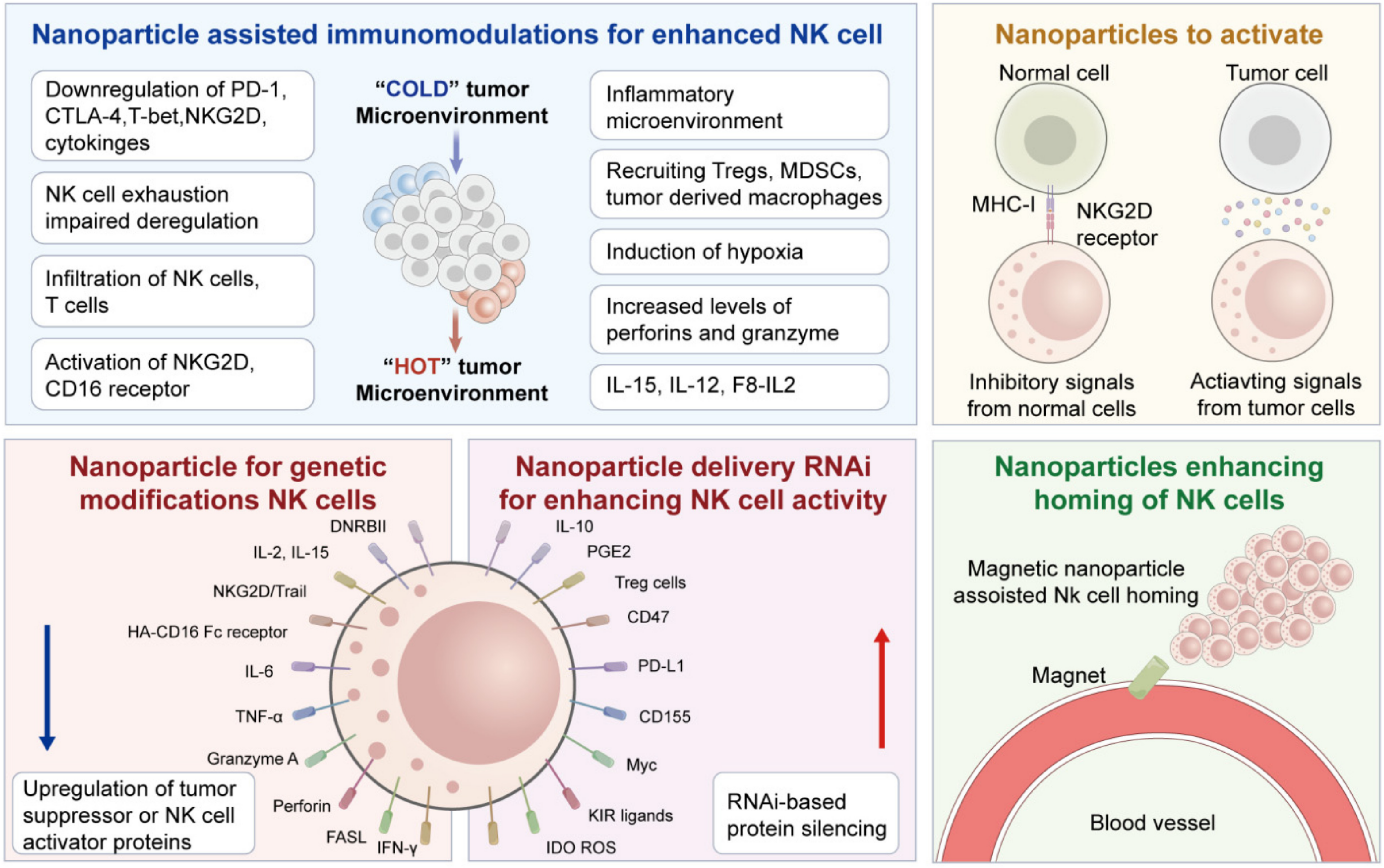
Details of various strategies for nanoparticles-assisted NK cell immunotherapies. Starting from the immunologic synapse with target tumor cells, resulting in the polarization and release of granzymes and Perforin. Figure redrawn from Murugan et al. (15) under the CC-BY license.

Beyond their direct catalytic activity, NK cells secrete cytokines like TNF-α and IFN-γ, which improve antigen presentation, promote robust T cell responses, and stimulate dendritic cells and macrophage activation (25). Figure 1 of the previous section (Introduction) shows these multi-layered effector functions whereas Figure 2 illustrates the immunologic synapse with target tumor cells, resulting in the polarization and release of granzymes and perforin. Clear engagement of death receptors, e.g., TNF-related apoptosis-inducing ligand (TRAIL) and Fas-Ligand (FASL), initiating apoptosis is evident in the figure. Images also show inhibitory signals (control cytotoxic results, KIRs binding to MHC-I), and activating cues (NKG2D-MICA/B interaction) (15)

### 2.2 Receptor-assisted regulation of NK cell activity

The interplay between inhibitory and activating receptors finely tunes NK cell function. Inhibitory receptors, including killer-cell immunoglobulin-like receptors (KIRs) and CD94/NKG2A, recognize MHC Class-I molecules on healthy cells and transmit signals to avoid autoimmune attack. On the other hand, activating receptors such as NKp30, NKG2D, NKp46, and NKp44 identify stress-induced ligands on infected cells, initiating cytotoxicity (26). GI tumors commonly activate this receptor balance to avoid immune surveillance (27). Additionally, immunosuppressive cytokines, like prostaglandin E2, TGF-β, and interleukin-10 (IL-10), lead to reduced NK receptor expression by disturbing cellular metabolism (28, 29).

Checkpoint blockade with anti-NKG2A antibodies or anti-KIR and administering designed ligands or recombinant cytokines are therapeutic strategies to restore NK activity. This receptor also enables the development of CAR-NK therapies, which introduce synthetic constructs without disturbing the functions of the cells’ native cytotoxicity (30).

### 2.3 Barriers to NK cells infiltration and endurance in GI tumor microenvironment

GI tumor microenvironment results in multiple biochemical limitations, restricting NK cell infiltration and persistence (31). Desmoplastic stroma and fibrosis severely restrict immune cell infiltration, especially in pancreatic tumors. Elevated interstitial fluid pressure and irregular vasculature further limit immune cell movement, while the dense extracellular matrix (ECM) damages trans-endothelial migration. The chemokine mismatch also undermines NK homing. Although GI tumors may secrete CXCL9/10/11 or CXCL12, infused NK cells usually lag behind the corresponding CXCR3 or CXCR4 receptors, decreasing chemotactic response. Additionally, the metabolic background of tumors, including acidosis, hypoxia, and glucose competition, reduces NK mitochondrial function and compromises cytolytic ability (32).

To further support the above discussion, Table 2 outlines some barriers to NK cell infiltration and durability in the GI tumor microenvironment and presents the corresponding nano-technological or immunological intervention strategies (33).

**TABLE 2:** Barriers to NK cell Infiltration and survival in the GI tumor microenvironment and potential interventions.

Barrier type	Description	Intervention strategy
**Physical barrier**	Dense ECM, abnormal vasculature, elevated IFP	ECM-degrading enzymes, magnetic-guided nanoparticles
**Chemokine mismatch**	Discrepancy between chemokine secretion and NK receptor profile	Chemokine-functionalized nanoparticles (e.g., CXCL10-loaded)
**Hypoxia and acidosis**	Disrupts energy metabolism and granule release	pH-responsive or oxygen-releasing nanocarriers
**Immunosuppressive cytokines**	TGF-β and IL-10 inhibit receptor expression and cytotoxicity	TGF-β inhibitors, immune checkpoint blockade via nanoparticles
**Metabolic competition**	Nutrient deprivation impairs NK viability and function	Metabolic reprogramming, glucose transporter mimetics

### 2.4 Emerging strategies to enhance NK cell therapeutic efficacy

Several strategies are developed to augment NK cell function in GI tumors and counteract these suppressive elements. Cytokine supplementation using IL-2, IL-15, or IL-21 enhances NK proliferation and cytotoxicity. Among these, IL-15 super-agonists and continuous-release nanoparticle formulations enhance NK cell activity and persistence *in vivo* (34). Barrier blockade signifies another encouraging avenue. PD-1, NKG2A, and TIGIT inhibitors can reverse NK cell exhaustion and restore granule exocytosis. Nanoparticles can be employed for the localized delivery of these inhibitors to mitigate systemic toxicity (35).

Adoptive cell therapy using CAR-engineered NK cells is gaining clinical momentum. These cells express artificial receptors targeting tumor-specific antigens while maintaining innate cytotoxic potential. Remarkably, CAR-NK cells can respond to tumor variants lacking the targeted antigen, giving an edge over the CAR-T cell therapies (36). As emphasized in Table 2, these enhancement strategies are critical for transforming the immunosuppressive TME into one that supports immune-mediated tumor eradication. Their integration with nanotechnology, which is explored in subsequent sections, constitutes a synergistic approach for next-generation NK-NP-based immunotherapy (37).

## 3 NK cell-based therapies in gastrointestinal tumors

Natural Killer (NK) cells are increasingly recognized as pivotal players in gastrointestinal (GI) tumor immune-surveillance. Their innate cytotoxic capability, unrestricted by antigen presentation, allows rapid identification and elimination of malignant cells, forming a compelling foundation for cancer immunotherapy (38). Nonetheless, the immunosuppressive TME, inefficient NK cell trafficking, and limited *in vivo* persistence hinder their therapeutic efficacy in GI malignancies (39). To address these challenges, various NK cell-based strategies, including autologous and allogeneic transfers, cytokine-induced memory-like NK cells, and CAR-engineered NK constructs, are under extensive investigation in both preclinical and clinical contexts (40).

### 3.1 Role of NK cells in GI tumor immunosurveillance

NK cells detect and eliminate transformed cells through recognition of stress ligands (e.g., MICA/B, ULBPs) and loss of MHC class I expression, common features of GI tumor cells. Their cytotoxic mechanisms include both granule-mediated (perforin and granzymes) and receptor-mediated apoptosis (FasL and TRAIL) (21). However, in GI tumors, this function is frequently suppressed by the immunosuppressive milieu, which upregulates inhibitory ligands such as PD-L1 and HLA-E and secretes suppressive cytokines including TGF-β and IL-10. These signals dampen NK activation and cytokine secretion, facilitating immune evasion and tumor progression (41). Therefore, restoring NK cell responsiveness within the hostile GI TME is a central therapeutic goal.

### 3.2 NK cell therapies: autologous vs. allogeneic approaches

Natural killer cell-based therapeutic applications are classified into autologous and allogeneic (42). Autologous NK cells therapy uses NK cells separated from the patient’s peripheral blood mononuclear cells (PBMCs), expanded *ex vivo*, and finally reinfused. While this strategy avoids graft-versus-host disease (GVHD), the NK cells may remain dysfunctional due to prior TME exposure, resulting in a limitation of their cytotoxicity (43). Allogeneic NK cells, collected from healthy donors, are not subject to tumor-induced suppression and may harness MHC mismatch to exert potent antitumor responses. However, this methodology often requires immunosuppressive or lymphodepletion conditioning and carries a potential GVHD risk (42).

**FIGURE 3:**
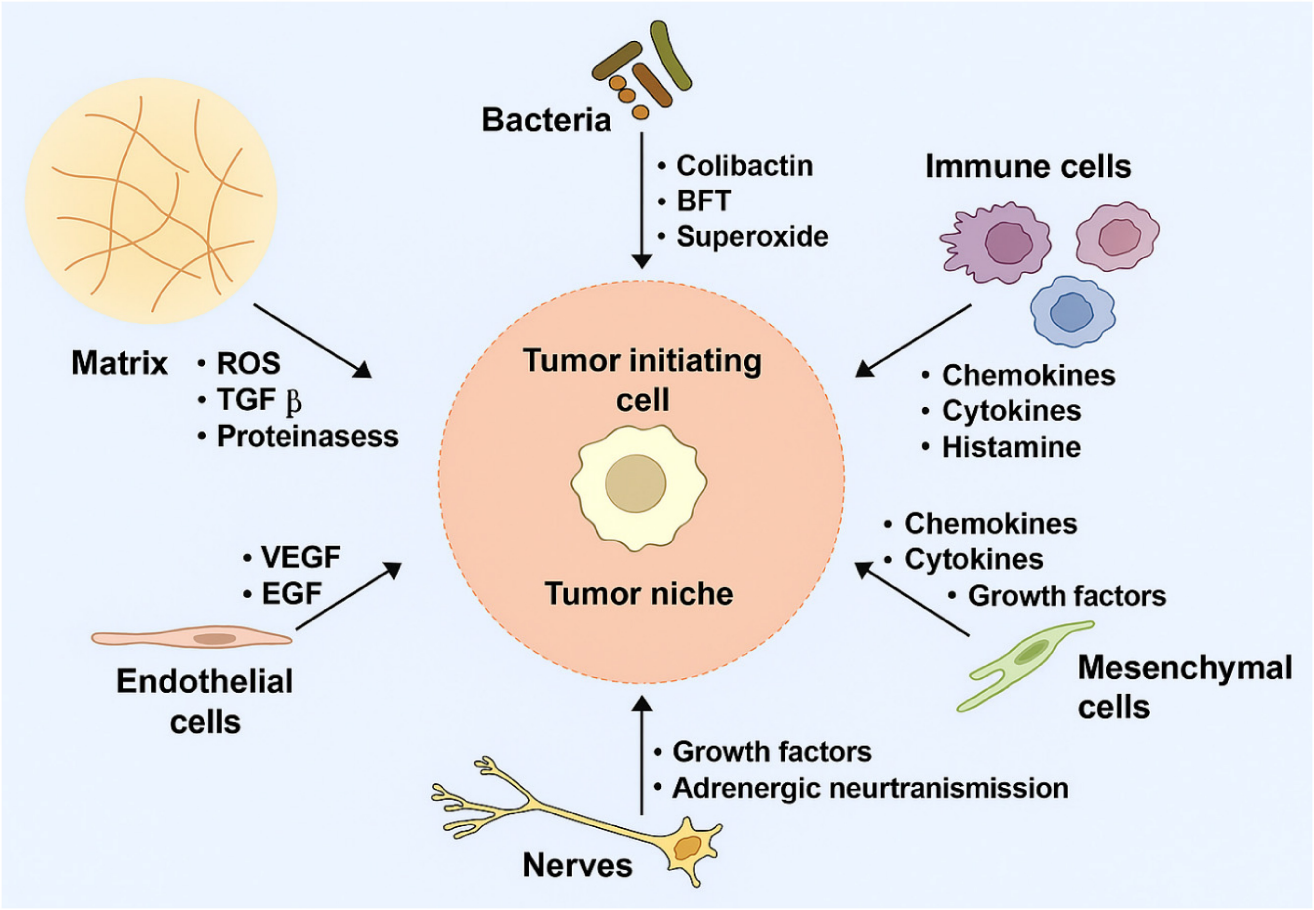
Tumor-initiating cell niches promoting proliferation and survival through cytokine signaling, epigenetic alterations, and chronic inflammation.

Another more advanced strategy is genetically engineering NK cells to express CARs. These synthetic constructs redirect NK cells toward tumor-specific antigens, enhancing precision and potency. In contrast to CAR-T cells, CAR-NK cells retain innate cytotoxic functions and pose a lower risk of cytokine release syndrome and GVHD (44). Figure 3 illustrates a typical CAR-NK architecture and the evolution of intracellular signaling domain designs (45). It has been shown that various types of cells in the (cancer) tumor microenvironment which produce tumorigenic cytokines and chemokines likely form the niche for tumor-initiating cells, or CSCs. These develop a proliferative environment which enables tumor-initiating cells to escape proliferate and apoptosis despite they need genetic alterations. The niche cells likely alter epithelial cells’ epigenetic regulation and signaling pathways. Moreover, the microbiota on the luminal side of the epithelium also contributes to the niche and is responsible for the maintenance of chronic inflammation (45, 46).

### 3.3 Clinical trials and emerging NK-based platforms in GI oncology

Several clinical trials are translating NK-based platforms into therapies for GI cancers. For example, a Phase I study (NCT04220684) using cord blood–derived NK cells for colorectal liver metastases demonstrated favorable safety profiles and partial clinical responses, underscoring the feasibility of NK-based cell therapy in solid tumors (47). Other clinical studies focus on enhancing antibody dependent cellular cytotoxicity (ADCC) through the combination of NK cells with monoclonal antibodies such as cetuximab or trastuzumab, particularly in HER2-positive gastric cancers (48).

Furthermore, cytokine-induced memory-like (CIML) NK cells, reactivated using IL-12, IL-15, and IL-18, show enhanced cytotoxic recall and persistence. They are used for pancreatic and colorectal tumor and show *in vivo* proliferation and robust IFN-γ production (49, 50). Universal NK strategies are being adopted using cell lines to address the challenges of person-specific cell therapies effectively. These strategies strengthen standardization, although challenges such as the limited therapeutic persistence in irradiated NK-92 cells are present (51). These developments of utilizing nanotechnology-assisted K cells herald a new era of precise immunotherapy for gastrointestinal cancers (52).

### 3.4 Summary of NK cell-based therapies in GI tumors

Table 3 summarizes NK cell-based therapeutic plans in GI cancers, including various therapeutic strategies for the role of NK cells in gastrointestinal tumors, such as colorectal, gastric, pancreatic, and liver cancers.

**TABLE 3:** Overview of NK cell-based therapeutic plans in gastrointestinal cancers.

Therapeutic strategy	Cancer type	Mechanism	Clinical stage*	Key notes
Adoptive NK cell transfer	Colorectal, gastric	Infusion of activated or expanded NK cells	Phase I/II	Often combined with cytokines or chemo
CAR-NK cells	Pancreatic, Liver	NK cells engineered with chimeric antigen receptors	Preclinical/early trials	Safer than CAR-T, promising targeting
Cytokine-based therapies	All GI cancers	IL-2, IL-15 to boost NK cell activation	Clinical/preclinical	Risk of systemic toxicity with IL-2
Checkpoint inhibition (e.g., anti-NKG2A)	Liver, colorectal	Blocking NK cell inhibitory receptors to enhance function	Phase I/II	Combination with PD-1 inhibitors under investigation
NK cell engagers (BiKEs/TriKEs)	Gastric, colorectal	Bispecific/trispecific antibodies linking NK cells to tumor antigens	Preclinical	Enhances cytotoxicity without genetic engineering
NK cell-derived exosomes	Pancreatic, Liver	Use of exosome-loaded cytotoxic molecules from NK cells	Experimental	Emerging field with potential for low-toxicity therapy
**NK cell lines (e.g., NK-92)**	Immortalized cell lines	Standardized cytotoxicity	Off-the-shelf, rapid expansion	Needs irradiation, limited persistence *in vivo*

*Database: https://db.dxy.cn/v5/home.

## 4 Synergy between NK cells and nanoparticles

For treating GI cancer, nanotechnology-assisted natural-killer cell based immunotherapy exhibits an advanced approach. Nanoparticles can help enhance NK cell activation and infiltration and develop prolonged cytotoxic activities. The standardization of NK cell preparation and the scalability of production remain challenges. Current methods for isolating and expanding NK cells are labor-intensive and variable, which can affect the consistency and efficacy of immunotherapy. Advancements in cell culture technologies and automation are needed to streamline NK cell production and ensure the reliability of therapeutic outcomes. In addition, the long-term safety and efficacy of combining nanoparticles with NK cells for immunotherapy have not been fully established. Long-term follow-up studies are required to assess potential adverse effects and to monitor the durability of therapeutic responses. Future research directions in this field include the development of novel nanomaterials and delivery systems that can enhance NK cell activation, infiltration, and persistence in tumor tissues. These nanomaterials should be designed to target specific markers on tumor cells and to deliver therapeutic payloads, such as cytokines or genes that can further stimulate NK cell activity. Furthermore, there is a need for multidisciplinary collaboration to integrate insights from immunology, nanotechnology, and oncology to develop comprehensive and personalized immunotherapy strategies. By combining expertise from these fields, researchers can address the complex challenges of treating gastrointestinal cancers and other solid tumors with NK cell-based immunotherapy.This section evaluates the mechanisms of action, design methodologies, and therapeutic potential of integrating nanoparticles with NK cells for precise drug delivery and treatment of gastrointestinal cancers.

### 4.1 Rationale for NK-nanoparticle integration in GI cancers

Due to elevated interstitial fluid pressure, a dense stromal matrix, limited immune cell infiltration, and immunosuppressive cytokine networks, gastrointestinal tumors are highly difficult to treat. NK cells often fail to accumulate at tumor sites as a result of chronic antigen exposure and metabolic stress, although they have intrinsic cytotoxic capabilities (53). NP-based drug carriers are capable of addressing these challenges. Functionalized NP surfaces increase immune evasion and tumor specificity (15). This integrated approach reprograms the TME, enabling more effective and precise immune responses (54).

### 4.2 Nanoparticles for sustained cytokine delivery and NK activation

Cytokines, such as IL-2, IL-15, and IL-21, are essential for NK cell expansion, effector function, and persistence. However, their systemic delivery is limited by these cytokines’ rapid degradation and toxicity (34). Biodegradable nanoparticles such as PLGA, chitosan-based hydrogels, and liposomes have been used to encapsulate these cytokines and release them at cancerous sites. In preclinical research, it has been shown that PLGA nanoparticles loaded with IL-15 significantly increase IFN-γ production and NK cell infiltration, which restricts tumor progression. Similarly, multi-delivery nanocarrier systems loaded with NKG2D and IL-21 ligands have been shown to enhance NK cell proliferation, resulting in the regression of tumors (55). Optimized system formulation boosts immune activation, enhances cytokine stability, and increases cellular uptake, reducing the site effects at the healthy cells within the tumor and its neighboring area. Enhanced formulation improves cytokine stability, immune activation, and promotes cellular uptake, while systemic adverse side effects are minimized (37).

### 4.3 Enhancing NK cell homing and tumor infiltration with nanoparticles

**FIGURE 4:**
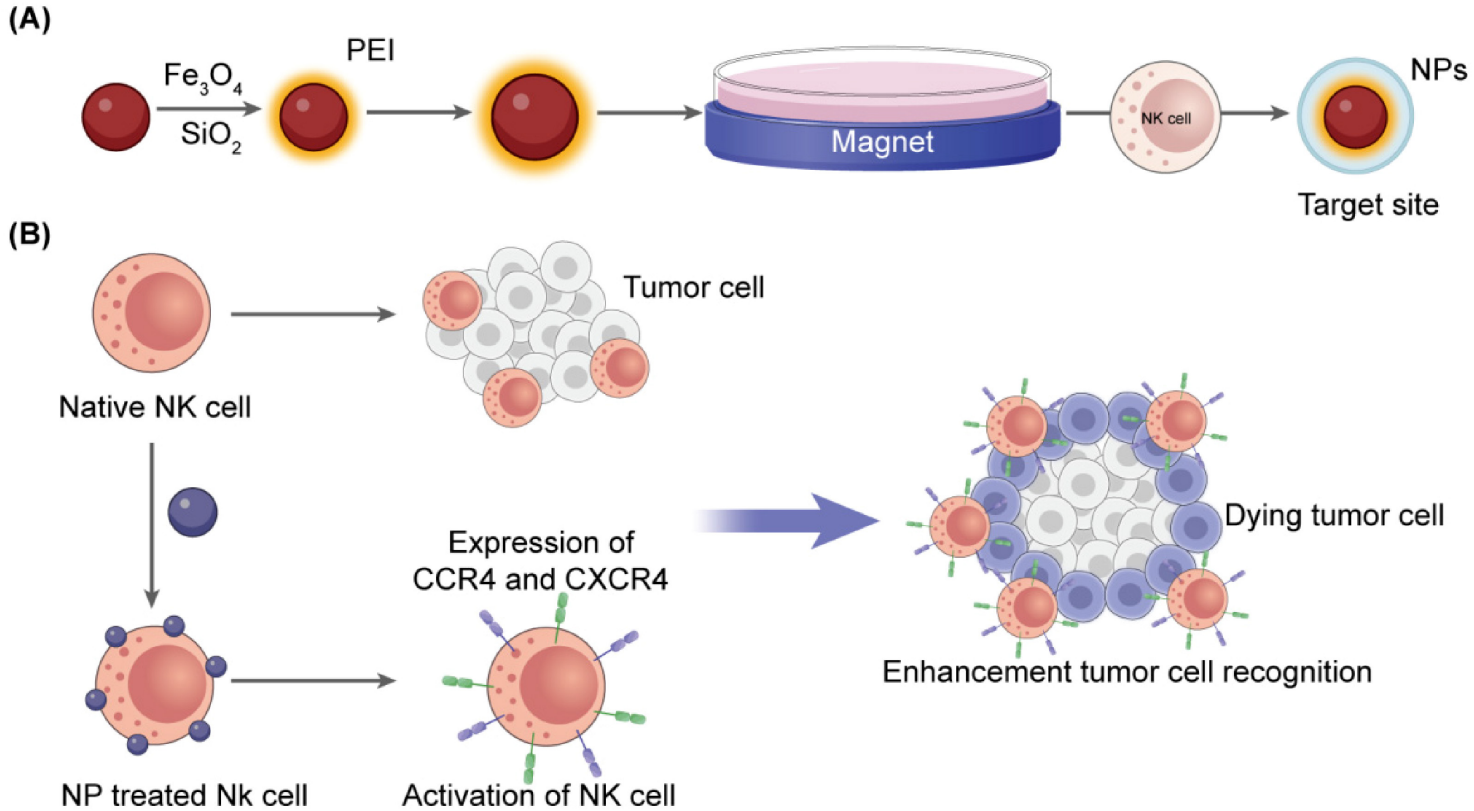
Schematic representation of NP-assisted activation of natural killer cells. **(A)** Fe_3_O_4_/SiO_2_ nanoparticles internalized by NK-92MI cells enable magnetic guidance and increase tumor infiltration (57). **(B)** Cationic magnetic nanoparticles upregulate CXCR4 and CCR4, enhancing directional migration and chemotactic response (60).

The ability of NK cells to persist and infiltrate within the tumor microenvironment strongly influences the effectiveness of immunotherapy. Drug delivery systems must be properly designed to enhance homing by incorporating chemokines like CCL5 or CXCL10, which generate chemotactic gradients that promote NK cell recruitment. In the case of colon cancer, CXCL10-loaded micelles have been shown to enhance directional NK cell migration and granule release (56). Magnetically active nanoparticles, such as oxides and ferrites, also enable external control of NK cells to reach the effective tumor environment. When coupled to NK cells, superparamagnetic iron oxide nanoparticles allow magnetic field-guided transport. The application of such nanoparticles resulted in the 17-fold enhancement of NK cell infiltration in colorectal tumor models (57, 58). Functionalization with endothelial adhesion molecules further improved their transmigration and vascular adhesion (59). Figure 4 demonstrates a schematic of NK-coated silica NPs delivering TRAIL and doxorubicin. The figure shows Fe_3_O_4_/SiO_2_ nanoparticles internalized by NK-92MI cells enable magnetic-driven, considerably enhancing tumor infiltration, chemotactic response, and directional migration.

### 4.4 Biomimetic and NK membrane-coated nanoparticles for targeted immunotherapy

Biomimetic nanoparticles exhibit a novel targeted delivery strategy, especially those coated with NK cell membranes. These constructs inherit native receptors such as DNAM-1, LFA-1, and NKG2D, enabling ligand-specific tumor delivery (61). In colorectal and gastric cancer models, NK membrane–coated mesoporous silica nanoparticles loaded with TRAIL and doxorubicin demonstrated prolonged circulation, improved homing, and increased tumor cell killing. These systems selectively interacted with MICA/B-expressing tumor cells, minimizing off-target effects (62–64).

**FIGURE 5:**
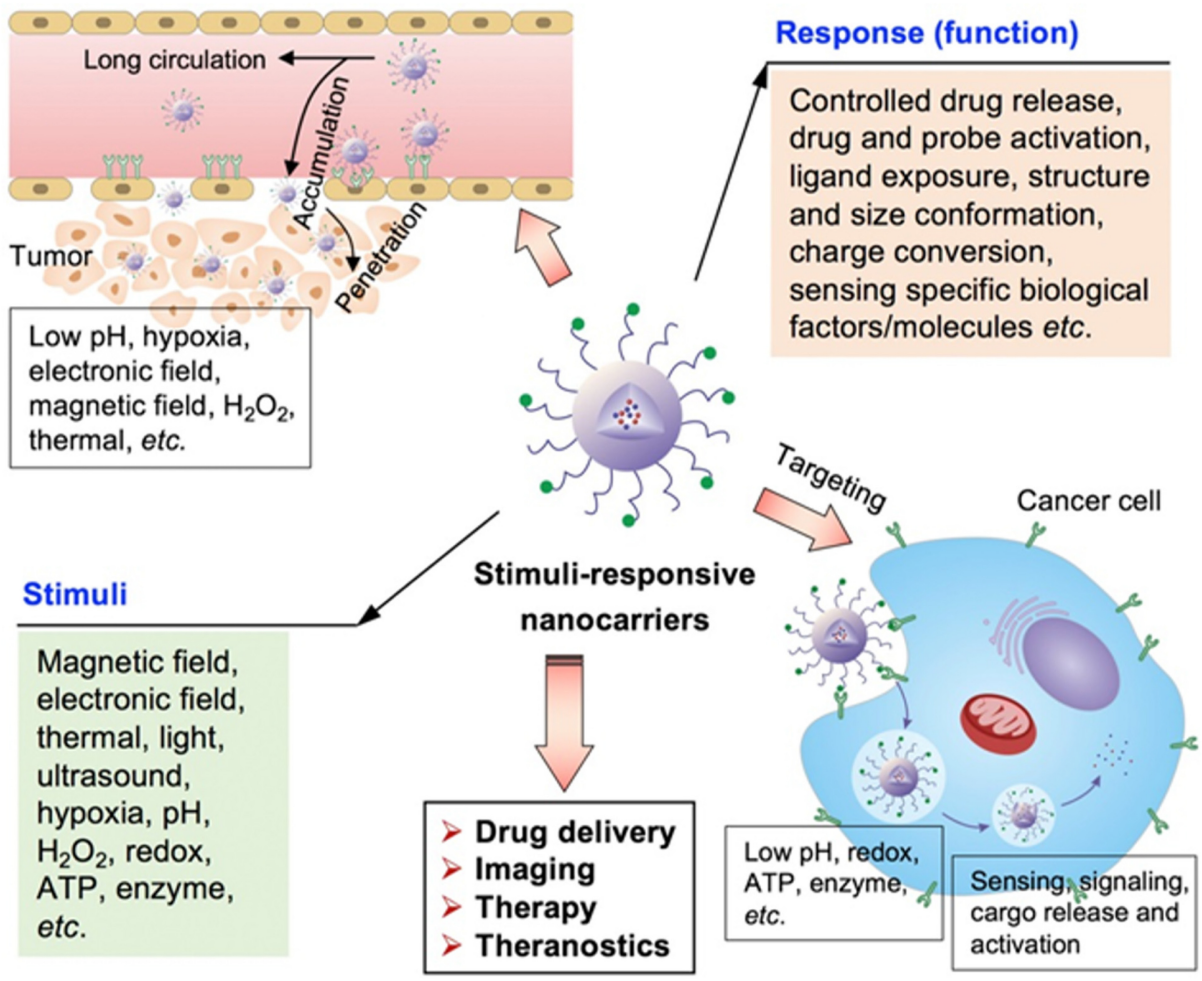
Schematic illustration of various stimuli-sensitive NPs engineered for precise drug delivery (65).

Figure 5 shows the stimuli-responsive nano-carriers (NPs) for drug transport to tumor sites. The drug carriers could penetrate and accumulate in tumors, and target the tumor cells to achieve different functions by responding to external and internal stimuli. To increase drug delivery specificity, the stimuli-responsive nano-carriers have been engineered keeping in view of different pathological profiles in intracellular compartments, normal tissues, and tumor microenvironment. These NPs could respond to external stimuli, such as the electric or magnetic fields, laser, ultrasound, or temperature. In addition, external stimuli could also influence the biological activities of these particles.

### 4.5 Combinatorial nanoparticles for immune checkpoint blockade and cytokine co-delivery

**FIGURE 6:**
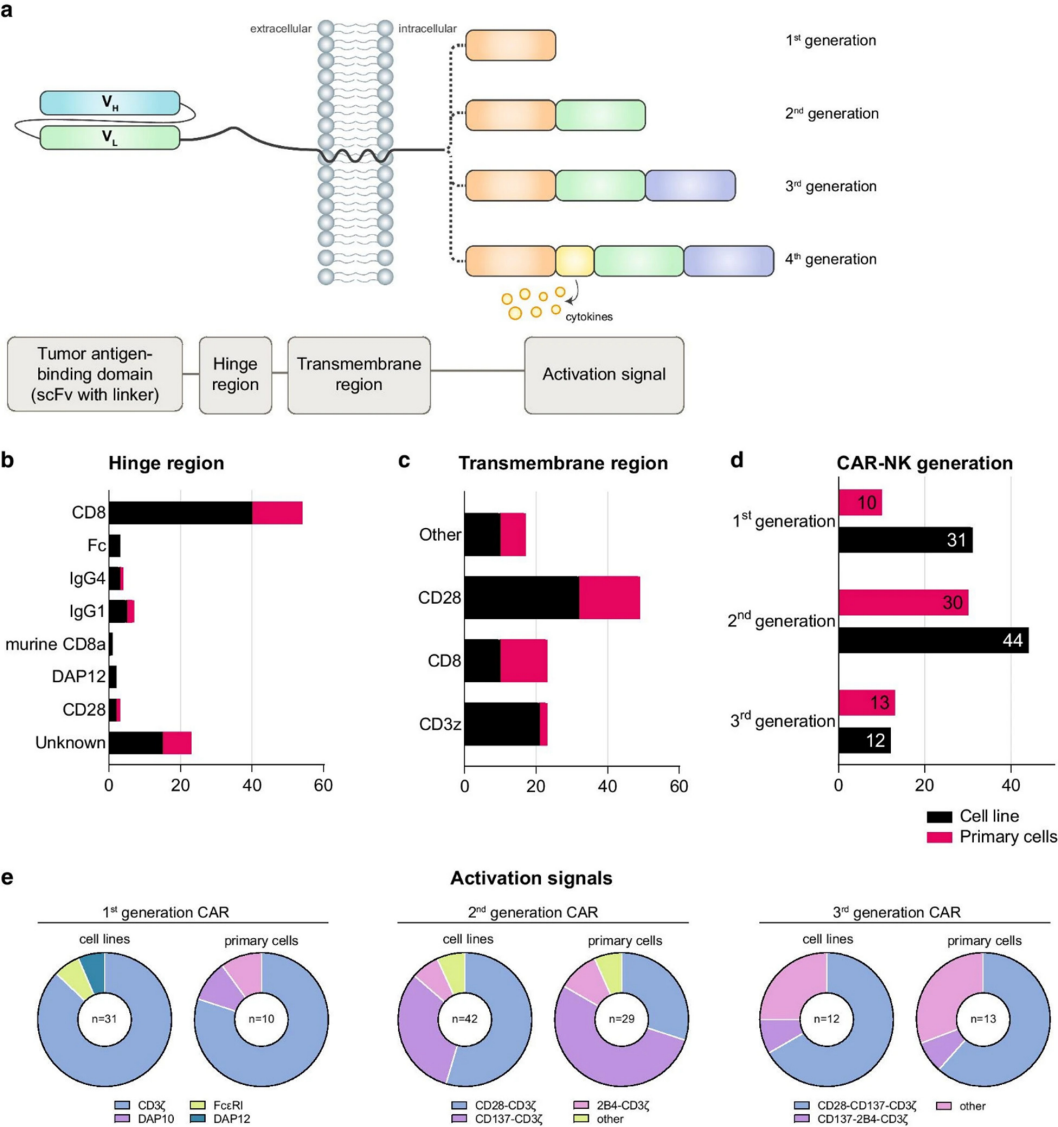
Structural design and functional components of CAR-NK cells across generations. **(a)** Modular CAR construct includes a tumor-targeting scFv, a flexible hinge, a transmembrane domain, and signaling domains (e.g., CD3ζ, 2B4, DAP10). **(b, c)** Statistical breakdown of hinge/transmembrane regions used in CAR-NK trials. **(d)** CAR generations employed across primary and cell line models. **(e)** Signal domain combinations used in each CAR generation. CAR-NK cells are increasingly combined with nanocarriers to co-deliver cytokines or immune checkpoint inhibitors, enhancing NK cell survival and function in the TME [redrawn from Gong et al. (70)].

Checkpoint molecules such as PD-1, TIGIT, and NKG2A contribute to NK cell dysfunction. Nanocarriers enable co-delivery of cytokines and checkpoint inhibitors, resulting in synergistic immune activation. For example, polymeric NPs co-loaded with IL-15 and anti-TIGIT antibodies restored NK cytotoxicity and IFN-γ secretion in gastric cancer models (66). These nano-platforms often incorporate pH-sensitive or enzyme-responsive release mechanisms to ensure spatial activation within the acidic TME. Multifunctional designs have also integrated glycolysis modulators or STING agonists to reshape tumor metabolism and promote NK cell activation (67–69). Figure 6 displays the structural composition of CAR-NK cells, highlighting particular domains’ functional roles. The hinge region adds flexibility, whereas the scFv defines target specificity. The transmembrane domain connects to intracellular signaling regions and anchors the CAR, determining the CAR generation. Fourth-generation CARs include cytokine-signaling elements. The figure provides valuable insight into the commonly used hinge and transmembrane domains, the distribution of CAR generations employed across studies, and preferred activation signals across CAR-NK cell sources.

### 4.6 Summary of synergistic strategies using NPs to enhance NK cell function

Table 4 outlines the various nanoparticles, their roles, the therapeutic objectives, and the results seen in preclinical research. These cooperative strategies lay the foundation for enhanced NK cell-based immunotherapy, capable of attaining enduring tumor management in gastrointestinal cancers.

**TABLE 4:** Details of application of nanoparticles to enhance NK cell function in GI tumors [adapted from Murugan et al. (15)].

Nanoparticle type	Function	Therapeutic target	Observed outcome
**PLGA, lipid-based, hybrid NPs**	Sustained cytokine delivery	IL-15, IL-21	Enhanced NK activation, proliferation, and tumor regression
**Superpara, chemokine-decorated NPs**	Tumor homing and navigation	CXCL10, CXCL12	Improved tumor localization, increased infiltration
**NK membrane-coated silica NPs**	Biomimetic immune cloaking	TRAIL, doxorubicin	Prolonged circulation, selective cytotoxicity
**Polymeric NPs with antibodies**	Checkpoint inhibition	TIGIT, NKG2A	Restored NK effector functions, reversal of exhaustion
**Hybrid lipid–chitosan nanocarriers**	Combined delivery platforms	IL-15 + anti-TIGIT	Co-activation of NK cells, reduced tumor volume

## 5 Modulation of the TIME

A significant barrier to the accomplishment of NK cell-based immunotherapy in GI tumors is the immunosuppressive nature of the TIME, which is composed of inhibitory cytokines, immunosuppressive cells, metabolic challenges, and stromal components. It has been shown that these factors hinder cytotoxic function, NK, survival, and cell infiltration. Their function is regularly impaired by the inhibitory ligands, suppressive cytokines, and competition for nutrients despite the fact that NK cells can reach the effective (tumor) sites successfully. Consequently, modulation (reprogramming) the TIME is important for achieving sustainable therapeutic results in NK cell-based therapies (71).

### 5.1 Immunosuppressive features of the TIME in GI tumors

Gastrointestinal tumors often display a immunosuppressive cytokine profile with the TIME, characterized by factors like TGF-β, IL-10, and VEGF characterized by factors such as TGF-β, VEGF, and IL-10. These factors suppress NK cell activity and hinder the activation of key receptors, including NKG2D, NKp30, and DNAM-1 (72). The cytokines, such as M2-polarized TAMs and Tregs, are secreted by the tumor cells and immunosuppressive populations. Furthermore, TAMs and MDSCs enhance immune suppression by releasing enzymes like indoleamine 2,3-dioxygenase (IDO) and arginase. These enzymes deplete tryptophan-amino acids and arginine, essential for NK cell metabolism and cytotoxic granule release (73). Simultaneously, chronic stimulation within the TIME leads to the upregulation of inhibitory receptors, including PD-1, TIGIT, TIM-3, and NKG2A, which promote NK cell exhaustion and diminish their cytotoxic potential (74).

**FIGURE 7:**
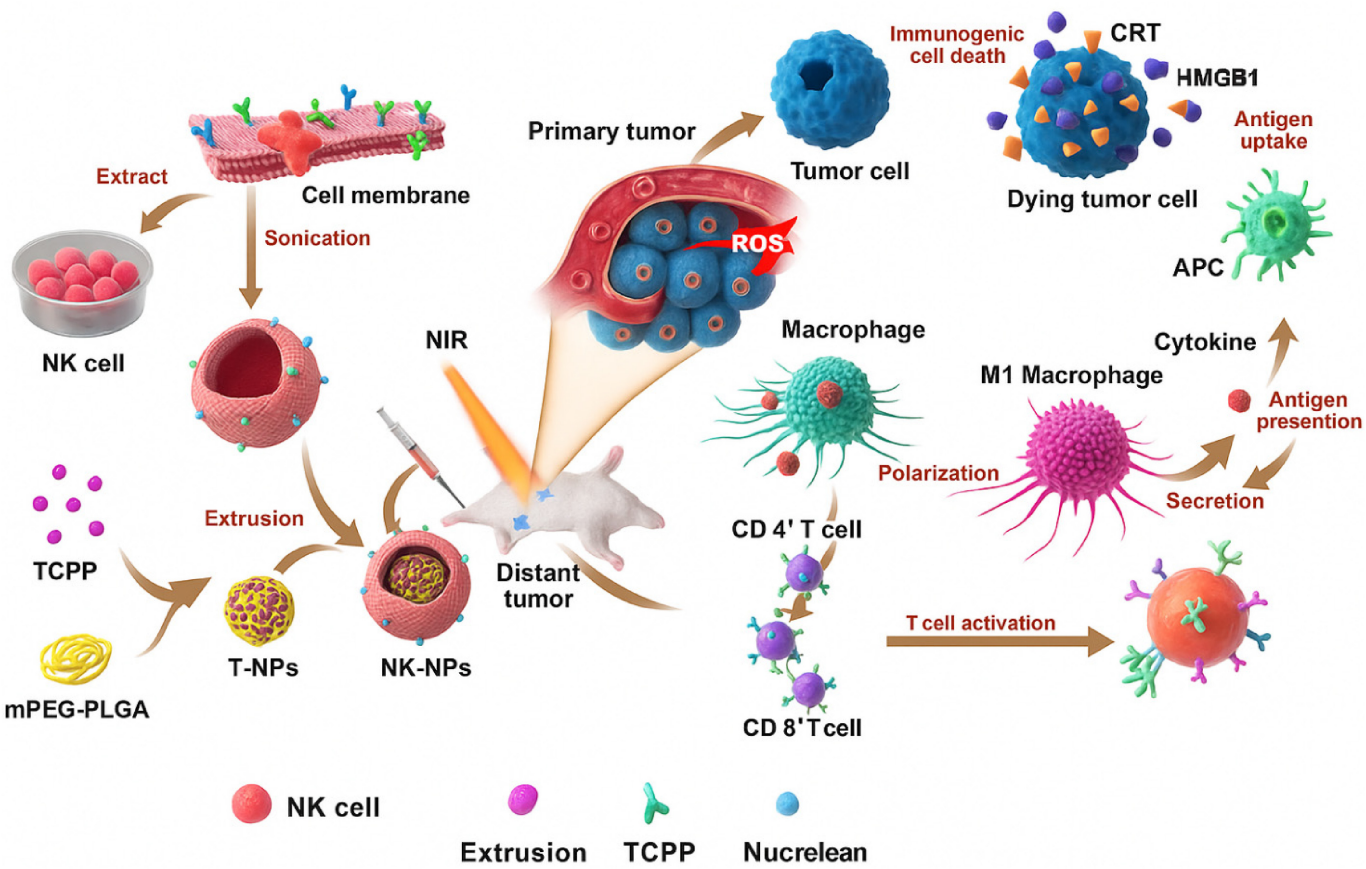
Representation of NK cell membrane-cloaked NPs for photodynamic therapy-improved cell membrane-based immunotherapy (77).

Metabolically, cancer cells rely heavily on aerobic glycolysis, consuming glucose and glutamine at high rates, depriving NK cells of the nutrients needed for oxidative phosphorylation and IFN-γ production (75). As illustrated in Figure 7, NK cell membrane-cloaked nanoparticles can enhance PDT-based immunotherapy (76).

**FIGURE 8:**
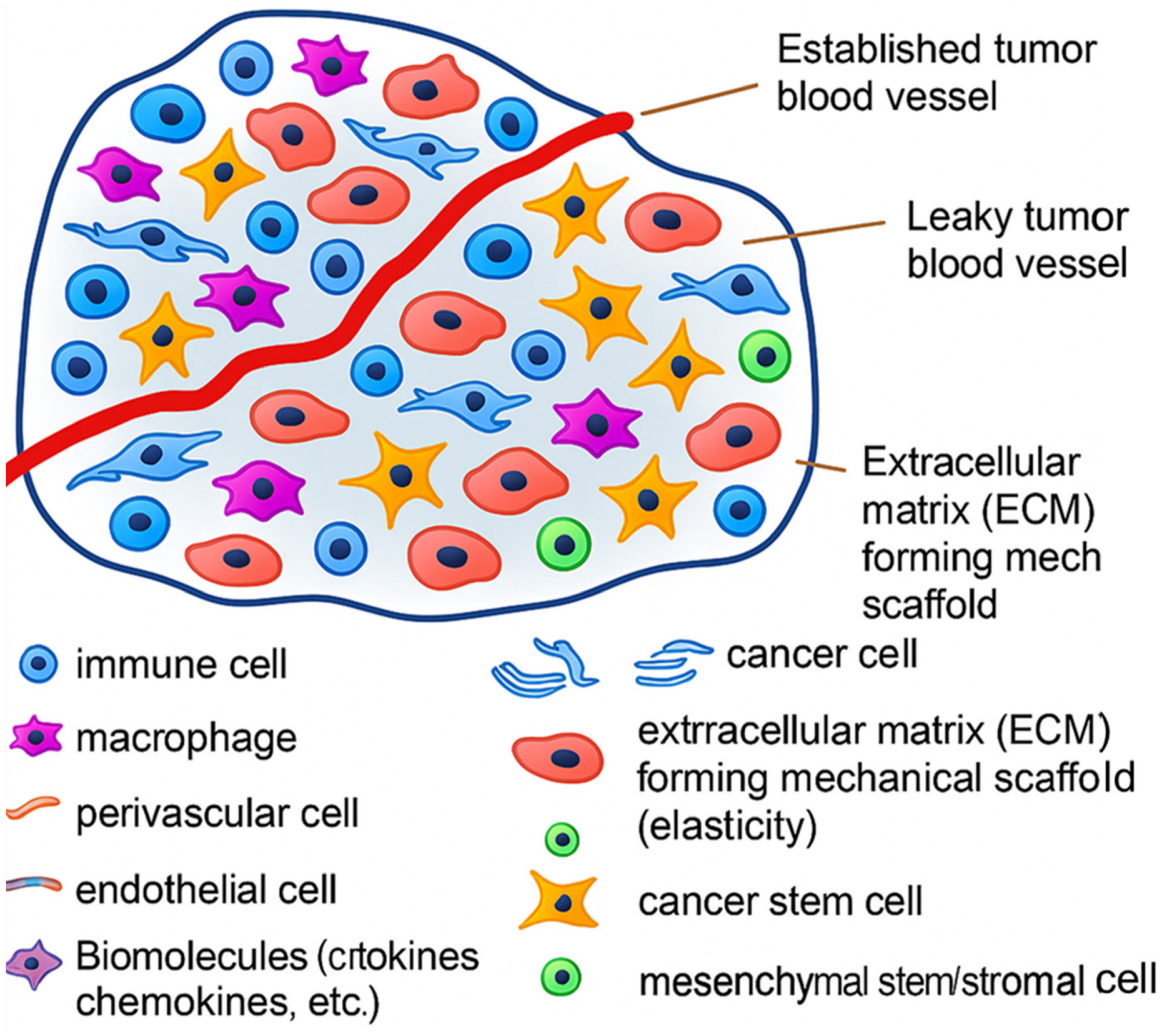
Structural elements of the tumor microenvironment in gastrointestinal tumors, inhibiting immune penetration and suppression of NK cytotoxic activity.

To further shed light on the Immunosuppressive Features of the TIME in GI Tumors, Figure 8 displays the structural elements of the TIME, including vasculature, fibroblasts, perivascular niches, and ECM. These elements create mechanical and biochemical limitations restricting immune cell function and infiltration (78).

### 5.2 Nanoparticle strategies for TIME reprogramming

Nanotechnology offers a useful platform for therapeutically reconditioning the TIME to allow the targeted delivery of cytokines, metabolic modulators, and immune checkpoint inhibitors directly to the cancerous tissue (79). Encapsulating IL-12, IL-15, or IL-21 in nanoparticles allows cytokine release at the tumor site, enhancing NK cell proliferation and cytotoxicity, while reducing cytokine toxicity (80). Nanoparticle distribution of STING or TLR agonists fosters dendritic cell maturation, activates IFN-I pathways, and repolarizes tumor macrophages to enhance innate immune cross-talk (69). Nanoparticles carrying anti-CSF-1R or chemotherapeutics can moderate TAMs and MDSCs, dropping immunosuppressive cytokines in the tumor microenvironment (81). Nanoparticle-conjugated antibodies, including anti-NKG2A and anti-TIGIT, restore IFN-γ secretion, enhancing checkpoint blockade and effective NK cell granular discharge (82). Gene-silencing nanoparticles that transport siRNAs targeting TGF-β or IDO can block suppressive signals and reactivate NK cell-mediated tumor elimination (23). Table 5 summarizes nanoparticle designs and their immunomodulatory effects on the GI tumor microenvironment (83).

**TABLE 5:** NPs-delivered immune-modulating agents for re-modulating the GI cancer immune microenvironment.

NP payload	Mechanism	Targeted outcome	NP type
**IL-12, IL-15, IL-21**	Activate NK cells, stimulate cytokine release	NK proliferation, increased IFN-γ	PLGA, liposomes
**STING agonists (e.g., cGAMP)**	DC–NK cross-talk via IFN-I	Activation of TAMs, enhanced innate immunity	Lipid NPs, micelles
**Anti-CSF-1R**	Deplete TAMs, inhibit TGF-β	M1 repolarization, immune infiltration	Polymeric NPs
**Anti-NKG2A, Anti-TIGIT**	Block NK cell inhibitory checkpoints	Restored cytotoxicity and degranulation	Liposomes, dendrimers
**TGF-β siRNA**	Downregulate immunosuppressive signaling	Reactivate NKG2D, promote NK function	Chitosan-based siRNA NPs

### 5.3 Enhancing intercellular immune crosstalk in the TIME

Modulating the tumor immune microenvironment comprises restoring cross-talk between NK cells and other effectors, such as immune-activating cells, macrophages, and Tumor-infiltrating T cells. Cytokines such as IFN-γ and GM-CSF secreted by NK cells enhance antigen presentation and DC maturation. At the same time, DC-derived IL-12 reciprocally establishes a synergistic feedback loop and activates NK cells (84). Nanoparticles co-loaded with agents, such as IL-15 and CD40L, have been demonstrated to simultaneously activate dendritic cells and NK cells, resulting in the enhancement of coordination between the intrinsic and adaptive immune responses (85, 86). Moreover, repolarizing M2 tumor-associated macrophages to M1 phenotypes via nanoparticle-encapsulated toll-like receptor (TLR) agonists or IFN-γ delivery confirms a pro-inflammatory milieu supportive of natural killer function (87).

Furthermore, nanoparticles delivering epigenetic or metabolic modulators can re-modulate the tumor immune microenvironment at a systems level, supporting the formation of immunological memory and promoting immune infiltration. These strategies are specifically important for immunologically “cold” gastrointestinal tumors, which are categorized by T cell exclusion and NK cell exhaustion (37). When integrated, these TIME-modifying nanotechnologies and NK-based therapies suggest significant potential for sustainable tumor control in treating gastrointestinal cancers (88).

## 6 Preclinical and clinical evidence

Extensive preclinical research has exhibited the high potential of integrating NK cells with nanotechnology in gastrointestinal tumor models. These models revealed improved NK cell survival and tumor infiltration, potential for remodeling the TIME, and increased antitumor ability. Such results support the possibility of translating NK-NP systems and place the foundation for clinical developments (37).

### 6.1 *In vivo* validation of NK-NP therapies in GI tumor models

Preclinical studies reveal that NK-NP platforms provided outstanding results in controlling tumors based on murine colorectal, gastric, and pancreatic cancer models. It has been seen that these systems increase NK cell infiltration, improve cytotoxicity, and extend immune activation within the tumor microenvironment. An example is the administration of IL–15–encapsulated PLGA nanoparticles in mice with subcutaneous colorectal tumors. The results led to almost a two-fold upturn in intratumoral NK cells, significant tumor regression, and high interferon-gamma (IFN-γ) secretion (89). In the same way, Tang et al. (90) presented another study revealing that NK cell membrane-coated silica nanoparticles co-loaded with TRAIL and doxorubicin for gastric tumor treatment. These studies demonstrate that biomimetic NPs targeted tumor accumulation, prolonged circulation, and potent NK-like cytotoxicity without systemic toxicity.

In another study, Wang et al. (91) proposed magnetically guided nanocarriers for targeted drug delivery and showed that this strategy was quite effective. They employed superparamagnetic iron oxide nanoparticles conjugated to NK cells and directed their movement using an external magnetic field. This methodology provided results of a 17-fold increase in tumor infiltration and a 60% decrease in tumor volume. This confirm that this approach is effective (57, 92). Table 6 summarizes these preclinical validations, highlighting nanoparticle types, cargoes, tumor models, and therapeutic outcomes for 2020–2025. The data affirm that multifunctional nanocarriers can potentiate NK cell-mediated antitumor immunity by enhancing delivery, functionality, and modulation of the TIME in GI cancers.

**TABLE 6:** Preclinical results of NK-nanoparticle therapies in gastrointestinal cancer models (survey period is 2020–2025).

Study (year)	Tumor model	Nanoparticle type	Cargo/strategy	Key outcome
**2020**	Colorectal (CT26)	PLGA NPs	IL-15 cytokine	↑ NK infiltration, ↑ IFN-γ, ↓ tumor growth
**2021**	Gastric (MKN-45)	NK membrane-coated silica NPs	TRAIL + doxorubicin	↑ tumor targeting, ↑ apoptosis, prolonged survival
**2021**	Pancreatic (Panc02)	Liposomes + anti-NKG2A	Checkpoint blockade	Reversal of NK exhaustion, improved survival
**2022**	Colorectal (HCT116)	SMNPs-conjugated NK cells	Magnetic guidance	17 × ↑ tumor localization, 60% ↓ tumor volume
**2023**	Gastric (AGS)	Hybrid lipid–polymer NPs	IL-21 + STING agonist	TAM repolarization, ↑ NK–DC cross-talk
**2024**	Pancreatic (KPC model)	Exosome-coated biodegradable NPs	IL-15SA + PD-L1 siRNA	> 70% tumor regression, ↓ MDSCs, ↑ NK persistence
**2024**	Colorectal (PDO + PDX)	mRNA-loaded lipid NPs	NK-activating ligands (NKG2D, MICA mRNA)	Robust NK activation, ↓ immunosuppression, enhanced tumor control *in vivo*
**2025**	Liver metastases (CRC)	Magnetic iron oxide NPs (MIONs)	TRAIL + homing peptides	Precision liver targeting, ↑ NK trafficking, prolonged survival in mice
**2025**	Gastric (3D Organoid)	Dual-targeted NPs (EpCAM + PD-L1)	IL-12 + anti-TIGIT nanobody	Synergistic immune activation, ↑ tumor necrosis, minimal off-target effects

### 6.2 Clinical trials and human applications

While the NK-NP design has yet to be implemented clinically, NK cell-based immunotherapies have advanced remarkably in GI cancer administration. Phase I and II clinical observations have explored the application of allogeneic NK cells, cytokine-induced memory-like (CIML) NK cells, and NK-92 cell lines in colorectal, pancreatic, and gastric cancers, with promising safety and efficacy profiles (93). For instance, a Phase I clinical trial (NCT03319459) involving CIML-NK cells in metastatic colorectal cancer reported increased IFN-γ levels and partial tumor regression without severe adverse effects (94). Another study (NCT04220684) applied cord blood–derived NK cells in patients with liver metastases of GI origin, demonstrating tumor shrinkage and absence of GVHD (95).

**FIGURE 9:**
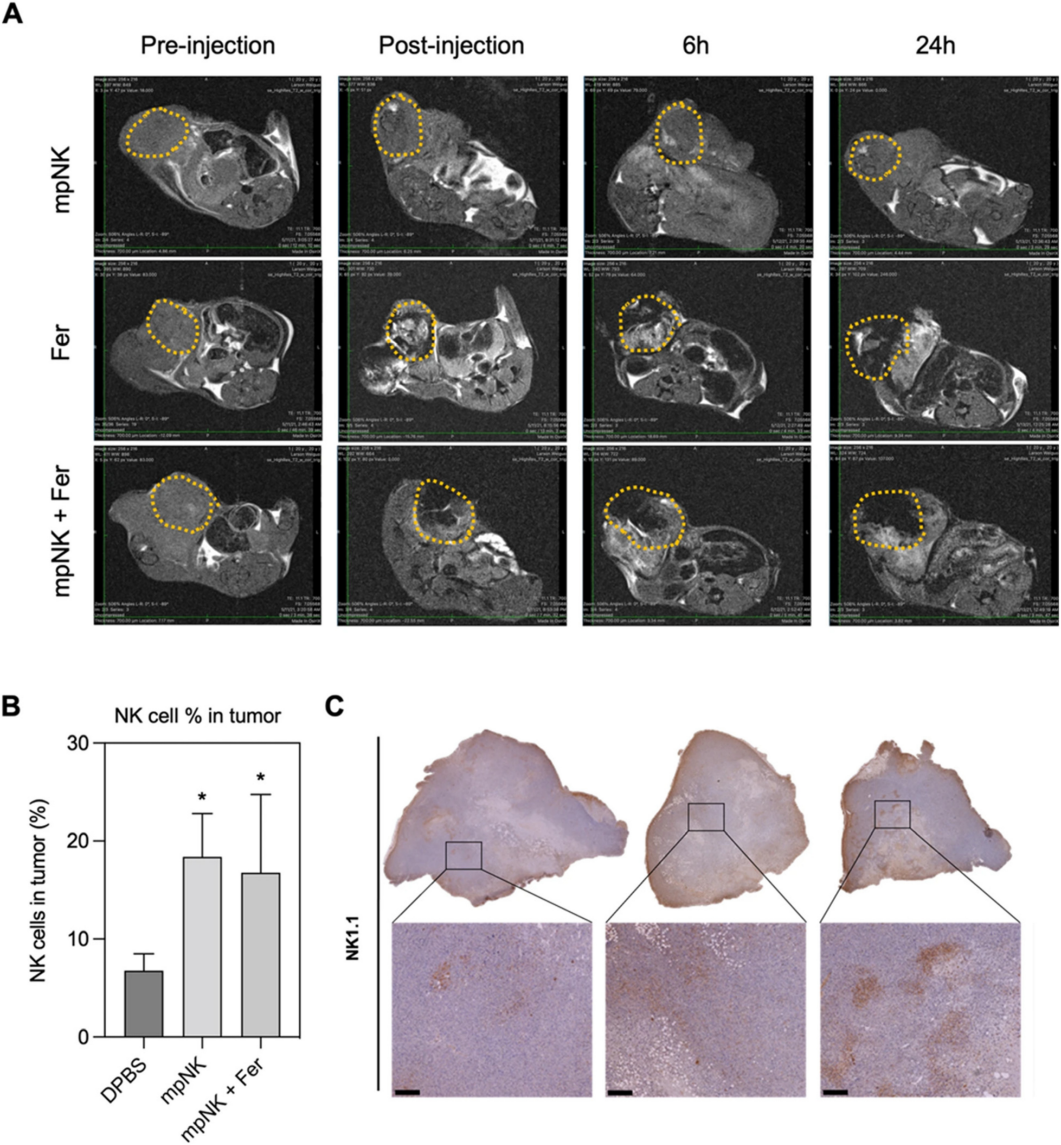
Translational roadmap for NK-NP therapies in gastrointestinal cancer. MRI T2 images of tumors treated with intra-tumoral injection with three combinations: only NK cells, ferumoxytol, and ferumoxytol and NK cells together **(A)**, flow-cytometry quantification of NK cells in cancer treated with NK cells, DPBS (Dulbecco’s Phosphate-Buffered Saline), or ferumoxytol and NK cells **(B)**, immunohistochemical images of NK-using the 1.1 for each group (scale bar = 100 μm) **(C)**. Figure reproduced from Kim et al. (98) under CC BY license. *P < 0.05.

In parallel, nanoparticle-based therapeutics have reached clinical use in GI cancers. For example, liposomal irinotecan (Onivyde^®^) is FDA-approved for metastatic pancreatic cancer, providing proof-of-concept for the clinical viability of nanocarrier-based delivery systems (96). Although no current clinical trial has combined NK cell therapies with nanoparticle systems, several early-phase trials investigate related combinations in solid tumors. CAR-NK cell therapies (e.g., NCT04324996 for HER2-positive tumors) and NP-mediated checkpoint inhibitor delivery systems are progressing toward human testing (97). The convergence of these modalities appears imminent as evidence accumulates on their complementary mechanisms. The developmental trajectory of NK-NP therapies is illustrated in Figure 9, which outlines key translational stages from preclinical design to regulatory approval. Research has focused on early translational phases, with clinical integration plans expected to be implemented within the next 5–7 years (98).

Although nanoparticle-based therapies such as liposomal irinotecan (Onivyde^®^) have achieved clinical application in gastrointestinal cancer treatment and demonstrated the clinical feasibility of nanocarrier delivery systems, patients’ concerns regarding the long-term safety and potential unknown risks of nanomaterials persist. These concerns may stem from insufficient understanding of nanomaterial biocompatibility, *in vivo* distribution, cumulative effects, and potential toxicity. Regarding long-term risks, there is currently insufficient data to comprehensively evaluate the safety of long-term nanomaterial use in humans. Patients may worry that nanomaterials could trigger chronic inflammatory responses, immune system interference, or unknown health issues. Therefore, long-term follow-up studies and safety assessments are crucial for building patient confidence. In terms of cost-effectiveness, the design and implementation of nanomaterial therapies such as NK-NP (natural killer cell-nanoparticle) may involve high research and development, production, and regulatory costs. These costs could ultimately be passed on to patients, increasing treatment expenses. Thus, while ensuring efficacy, reducing treatment costs and improving the cost-benefit ratio are key to the widespread adoption of nanomaterial therapies. As for patient eligibility criteria, clinical trials for nanomaterial therapies typically impose strict inclusion criteria to ensure study validity and safety. These criteria may include tumor type, stage, patient age, physical condition, prior treatment history, etc. Whether a patient meets these criteria directly determines their eligibility for nanomaterial therapy. Therefore, when establishing patient inclusion criteria, individual differences and practical needs should be fully considered to ensure treatment fairness and accessibility. In summary, by strengthening research, improving transparency, reducing costs, and refining patient eligibility criteria, the safe and effective application of nanomaterial therapies in gastrointestinal cancer and other fields can be advanced.

## 7 Roadmap for the future

The advancement of NK–NP synergistic therapies for gastrointestinal (GI) malignancies is accelerating, driven by promising preclinical data and mounting translational efforts. However, the transition from laboratory proof-of-concept to clinical implementation entails multifaceted challenges. A strategic roadmap that integrates considerations of technological scalability, clinical feasibility, regulatory oversight, and economic viability is imperative to transform these innovations into clinically viable oncology interventions (37).

### 7.1 Advancements in bioengineering and next-generation NK platforms

Future development in NK-NP therapeutics will be supported by innovations in bioengineering, logic-gated chimeric antigen receptor NK (CAR-NK) systems that will be based on nanomaterial designs. Presently, the nanoparticle platforms are being tailored to deliver multiplexed payloads, ranging from cytokines and immune checkpoint inhibitors to gene editors and imaging agents, enabling integrated theranostic applications (99). Genetic modification practices enhance NK cell capabilities by enhancing the expression of chemokine receptors or suppressing inhibitory receptors (e.g., CXCR2). With the help of nanotechnology, these modifications can be restricted to tumor environments, improving their safety and precision (100). Together, the development of GMP-compliant bioreactor designs for NK cell expansion and automation strategies for nanoparticle preparation is addressing the demand for scalable and reproducible clinical-grade production (101).

### 7.2 Overcoming regulatory and clinical translation barriers

Although there is strong preclinical support, several factors still prevent the translation of NK–NP platforms to clinical applications. These factors include complicated nanoparticle formulation characterization, establishing pharmacokinetics and toxicity, and maintaining batch-wise quality control (102). Regulatory bodies such as the FDA and EMA require detailed physicochemical characterization, such as size, zeta potential, and encapsulation efficiency, as well as functional validation of cell-based components through cytotoxicity assays and cytokine release profiles (103).

Collaborative frameworks like the FDA INTERACT program have emerged to streamline this process, facilitating early dialogue between developers and regulators. Additionally, harmonization initiatives are proposed to standardize nanoparticle quality control and immune cell release criteria (104). Key barriers include selecting suitable tumor types for early-phase trials, developing biomarkers to stratify NK-sensitive patients, and integrating real-time immune monitoring platforms (105). Table 7 outlines the translational roadmap, summarizing the critical milestones, projected timelines, key challenges, and proposed solutions for NK–NP therapeutic deployment in GI cancers.

**TABLE 7:** Strategic roadmap for clinical translation of NK-NP therapies in GI cancers.

Milestone	Projected timeline	Key challenges	Proposed solutions
**Standardized NK-NP platform design**	2025–2027	Variability in NP properties	Modular NP scaffolds; automation-based quality control
**Preclinical-to-IND transition**	2026–2028	Unclear regulatory path for hybrid products	Early engagement with FDA/EMA; adaptive clinical designs
**Phase I/II trials in GI cancers**	2027–2029	Patient stratification and monitoring	Biomarker-guided cohorts; companion diagnostics
**GMP manufacturing scale-up**	2028–2030	Production cost and batch consistency	Closed-system bioreactors; robotic nanoparticle formulation
**Commercial launch (conditional)**	Post 2030	Reimbursement and regulatory harmonization	Health tech assessments: global consortium development

### 7.3 Integration with precision oncology and combination immunotherapies

NK–NP technologies are naturally aligned with the evolving precision oncology paradigm. Emerging genomic and transcriptomic platforms will likely identify GI tumor subtypes, particularly immune-cold tumors, that may benefit most from NK–NP immunomodulation (106). Moreover, these synergistic platforms are well-suited for integration with other therapies such as checkpoint blockade, CAR-T therapy, and microbiome-targeted immunotherapies (107).

Multimodal treatment strategies incorporating stimuli-responsive nanocarriers, tumor-targeting ligands, and personalized CAR-NK designs promise to create adaptable immune interventions. AI is increasingly used to optimize nanoparticle configurations, simulate tumor–immune interactions, and predict patient response trajectories (108). As the intersection of nanomedicine, immune engineering, and AI deepens, NK-NP platforms are poised to become foundational components of individualized cancer care (109).

### 7.4 Regulatory alignment and long-term safety monitoring

As hybrid systems, NK-NP therapies pose unique regulatory complexities that demand forward-looking frameworks. Regulatory agencies must define precise standards for nanoparticle characterization, including zeta potential, polydispersity index, and degradation kinetics. Functional validation of NK cells post-formulation, including cytolytic activity and phenotypic stability, remains essential for preclinical and clinical evaluation (110).

Moreover, real-time pharmacokinetic and pharmacodynamic monitoring must be incorporated into early-phase trials to refine dose optimization and immune stability. Long-term safety data must be gathered through structured post-marketing surveillance, particularly regarding nanoparticle accumulation in off-target tissues (111). Ethical risk management, developed collaboratively between academia, biotech firms, and regulators, will play a central role in ensuring the safe clinical adoption of these therapies (West Journal of Biology, Pharmacy and Health Science, n.d.).

### 7.5 Translational roadmap for NK–NP systems

**FIGURE 10:**
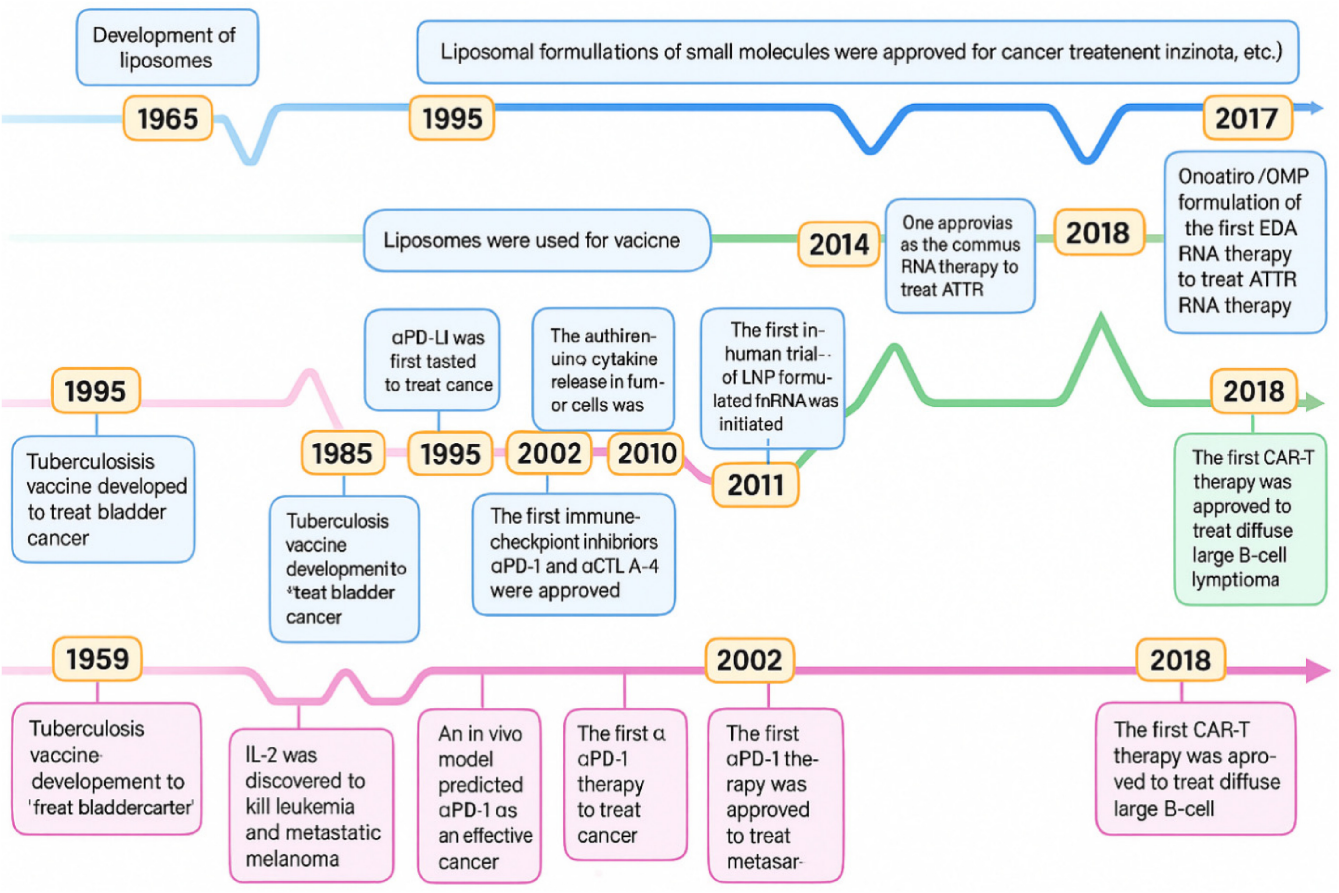
Time progression of the key milestones through developing cancer immunotherapies and lipid-based nanoparticles.

A visual roadmap (Figure 10) highlights the phased progression of lipid-based nanoparticle systems in cancer immunotherapy, from synthesis and preclinical assessment to clinical trials and regulatory approval. This timeline highlights the critical interplay between engineering innovation of nanotechnology, regulatory willingness, and immunological efficacy (112).

The successful clinical integration of NK cells and nanotechnology in GI cancers pivots on coordinated invention across medical and engineering disciplines. This includes cutting-edge nanomaterial designs, scalable good manufacturing practices, immune profiling for treatment, and coordinated global regulations. Following these roadmaps, NK-nanoparticle remedies can transform from experimental jurisdiction to standard clinical applications.

## 8 Conclusion

Integrating NK cell-based immunotherapy and nanotechnology suggests a transformative development in treating gastrointestinal cancer. This approach offers a sustainable and trustworthy solution to the problems associated with conventional treatments and standalone immunotherapies. This combined technology addresses critical challenges such as insufficient NK cell infiltration into the cancerous tissues; functional exhaustion induced by inhibitory signaling pathways, and restricted sustainability within the immunosuppressive TIME.

Specific designs of nanocarriers are coated with NK cell membranes, replicating natural cellular interactions within the tumor environment and further enhancing immune evasion. Nanoparticles enhance the absorption and biodistribution of therapeutic agents and offer accurate spatiotemporal control over their release, which enables localized modulation of the TIME. This well-defined delivery of therapeutic agents reduces the toxicity of the system and improves their treatment accuracy. Moreover, stimuli-responsive nanocarriers, sensitive to enzymatic activity, pH, or hypoxic variations, enable them to carry the accurate drug, release it at the tumor site, maintain a continuous NK activation, amplify tumor cell apoptosis, and foster long-term immunological memory.

Beyond direct cytotoxic enhancement, NK-NP platforms contribute to broader immune system reprogramming. These promote cross-talk between antigen-presenting and NK cells, like dendritic cells and macrophages, promoting a pro-inflammatory and immunostimulatory microenvironment. Such immune remodeling is particularly vital in GI tumors, which are often characterized by dense stromal architecture and immune-excluded phenotypes. By enabling immune cell infiltration and activation in these challenging landscapes, NK–NP therapeutics mark a paradigm shift toward precision-guided immune modulation. For multiple GI cancer models, the efficacy of NK–NP systems has been validated through different preclinical investigations. Initial clinical studies employing NK cell infusions, membrane-cloaked nanoparticles, and magnetically guided nanosystems have begun to demonstrate translational feasibility. Nevertheless, the clinical trajectory of these therapies will depend on overcoming key challenges, such as scalable and reproducible manufacturing, standardized regulatory frameworks, integration of real-time immune monitoring, and the development of patient-specific therapeutic algorithms.

Future progress will be catalyzed by integrating NK–NP platforms with multi-omics profiling, precision oncology, and artificial intelligence–driven design and prediction tools. This will enable the formulation of personalized NK–NP therapies that are precisely matched to an individual’s tumor immunogenomic landscape, potentially redefining standards of care for GI malignancies and extending applicability to other solid tumors.

In summary, the synergistic interplay between NK cell immunotherapy and nanotechnology offers a dynamic, modular, and highly adaptable platform for the next generation of cancer treatment. It holds immense promise for overcoming the multifaceted barriers of the tumor microenvironment and delivering personalized, effective, and less toxic immunotherapeutic solutions for gastrointestinal cancers and beyond.
